# Molecular and Clinical Considerations for Anesthesia in the Aging Brain

**DOI:** 10.3390/ijms262110272

**Published:** 2025-10-22

**Authors:** George-Abraam Tawfik, Michael Lu, Marc De La Hoz, William Crugnola, Zhaosheng Jin, Daryn Moller

**Affiliations:** 1 Department of Anesthesiology, Stony Brook University Hospital, Stony Brook, NY 11794, USAdaryn.moller@stonybrookmedicine.edu (D.M.); 2 Renaissance School of Medicine, Stony Brook University, Stony Brook, NY 11794, USA

**Keywords:** postoperative neurocognitive disorders, delirium, neuroinflammation, oxidative stress, synaptic dysfunction, glymphatic system, volatile anesthetics, dexmedetomidine

## Abstract

Postoperative neurocognitive disorders (PNDs) encompass a spectrum of cognitive dysfunction in the perioperative period. PNDs can present with hypoactive symptoms such as lethargy, hyperactive symptoms such as confusion and disorientation or a mix of both. PNDs can affect patients of all ages; however, the incidence of PNDs increases significantly as patients age. It is important to promptly recognize PNDs as patients can have higher morbidity and mortality, longer hospital stays, higher readmissions rates, and additional testing/treatment after discharge. In this review, we explore the molecular basis involved in brain aging as well as the mechanisms involved in anesthesia exposure and the development of PNDs. Understanding the mechanisms behind brain aging and the parallels to the pathophysiology of PNDs such as neuroinflammation, oxidative stress, mitochondrial dysfunction, and synaptic disruption are integral to mitigating the incidence and severity of PNDs. Current research suggests possible clinical targets for management such as dexmedetomidine and NSAIDs due to their abilities to combat harmful neuroinflammatory effects. Additionally, EEG-guided anesthesia, careful choice of anesthetics, and supportive measures can aid in mitigating PNDs. By understanding the mechanisms of brain aging, the risk factors for and pathophysiology of PNDs, we can better tailor our management of PNDs.

## 1. Introduction

Life expectancy continues to rise with ongoing advancements in medicine and technology. Based on the 2020 US census, roughly 1 in 6 adults was age 65 and over, and the total number of elderly worldwide is estimated to reach 1.5 billion by 2050 [1]. As the population ages, so do surgical patients. A global surgical outcomes study found the mean age of elective surgical patients to be 58 years old [2]. With increased age also comes an increase in a variety of intra- and postoperative complications. One such area of interest is postoperative cognitive dysfunction. There are growing data on the negative impact this dysfunction can have on patients, families, and healthcare systems. Notably, patients can experience higher morbidity and mortality, longer hospital stays, higher rates of readmission, and exposure to additional testing/treatment even after discharge [3]. In addition to the increased emotional and financial burden this places on patients and families, the resources needed to evaluate, treat, and follow up on patients suffering from postoperative cognitive dysfunction are estimated to cost 150 billion dollars in the United States annually [4].

**Figure 1:**
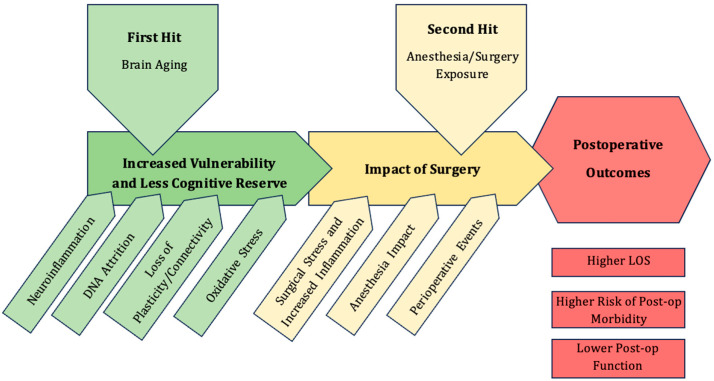
Two-hit model of PNDs, highlighting the pathophysiology of brain aging which reduces the cognitive reserve, the critical perioperative events and postoperative trajectory.

The development of PNDs can be considered in a “two-hit” model (Figure 1). Pre-existing patient vulnerabilities, when compounded by critical perioperative events, can result in the development of overt neurocognitive deficits. Aging, frailty and comorbidities all contribute to the decline in brain health and cognitive reserve. Similarly, perioperative exposure can adversely impact brain health and cognitive function. In this review, we will discuss the pathophysiology of brain aging as well as the effects of anesthesia exposure.

## 2. Postoperative Neurocognitive Disorders

### 2.1. Timeline of PNDs

A PND encompasses a spectrum of cognitive dysfunction in the perioperative period. Postoperative delirium (POD) is the most acute (within hours to days) and common presentation of PNDs, with an incidence anywhere from 10–50% [5]. Patients can experience hypoactive delirium, during which a patient can appear sluggish or withdrawn. The subtle presentation may lead some to disregard hypoactive delirium as generalized fatigue, and lead to an underestimation of its incidence. Alternatively, patients can experience hyperactive symptoms such as confusion, hallucinations, or disorientation [3]. Patients may also experience a combination of hypoactive and hyperactive symptoms known as mixed delirium [6]. Postoperative cognitive dysfunction (POCD) is a decline in cognition (i.e., deficits in concentration, memory, and executive function) that remains even after a patient has otherwise recovered from surgery. The term POCD can be further delineated into delayed neurocognitive recovery (dNCR) if cognitive dysfunction persists beyond the acute perioperative period (i.e., after discharge) but not past 30 days post-op, or major/minor postoperative neurocognitive dysfunction (NCD) if still present between 1 and 12 months [5]. Neurocognitive deficit which persists beyond one year are not included under the definition of PNDs and are denoted as major or minor neurocognitive dysfunction. Major NCD (dementia) is distinguished from minor NCD by the presence of impaired activities of daily living. PNDs have been associated with a 12 times increased risk of dementia, which can necessitate further diagnostic testing and follow-ups, increasing healthcare costs for both the patient and hospital systems [7].

### 2.2. Epidemiology of PNDs

Patients of all ages, even young healthy individuals, can experience postoperative cognitive dysfunction; however, the incidence of PNDs increases significantly with age. There is a discrepancy in the literature regarding the incidence of POD, with reports ranging from 10% to 50%. These variations are likely secondary to differences in the significance of surgical procedure, inherent patient characteristics, and frequency/method of delirium assessment [8]. The symptoms of hypoactive delirium are more subtle, and without validated delirium assessments, there is likely an underestimation of the incidence of POD [9]. The highest rates of POD are in the elderly population and occur more often in cardiac and major non-cardiac surgery [7]. For example, Shah et al. found the incidence of POD in head and neck surgery to be 12%, compared to 50% and 51% in major abdominal and cardiac surgery, respectively [10,11,12]. There are several other surgical risk factors for POD, namely increased surgical duration (or length of bypass), open surgery, emergency surgery, and need for blood transfusion [12,13,14]. Among patient factors increasing the risk of POD are increasing age, ASA class (≥3), COPD, diabetes, pre-existing cognitive impairment, male sex, and lower education status [15,16,17,18,19,20,21,22]. 

Numerous perioperative anesthetic factors have been analyzed for their effect on the incidence of POD. The traditional philosophy in anesthesia has been to avoid perioperative benzodiazepines in the elderly. This sentiment is supported by some of the older literature, showing an increased incidence of POD when benzodiazepines are used as an anesthetic pre-medication [23,24,25]. Alternatively, the newer literature is being published, challenging this idea and demonstrating that benzodiazepines do not increase incidence of POD, while decreasing incidence of patient awareness [26,27]. As previously discussed, the effects of postoperative cognitive dysfunction can extend far beyond the postoperative period, with lasting impacts on patients’ cognition and functional status. Evered et al. found the incidence of PNDs in older patients to be between 9.9 and 12.7% three months postoperatively [28]. Considering the importance of age as a risk factor for PNDs, it is important to explore the molecular basis by which brains age and how we may utilize that knowledge to treat or even prevent PNDs.

## 3. Molecular Biology Basis for Brain Aging

**Figure 2:**
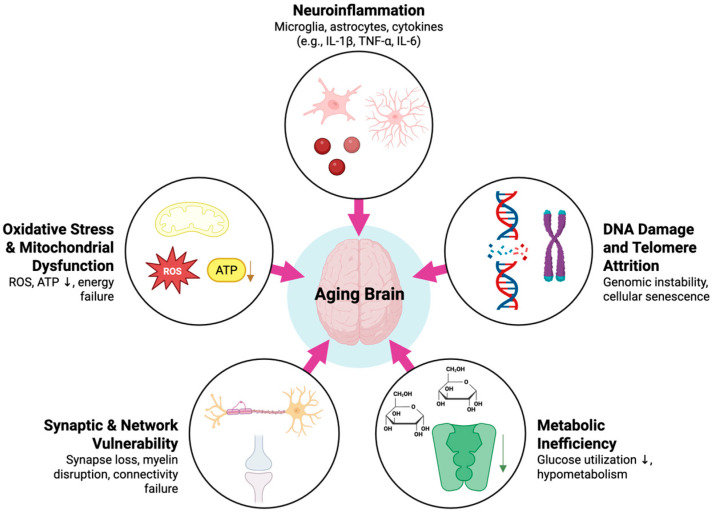
Molecular and Cellular Basis of the Aging Brain. Created in BioRender. Tawfik, G. (2025) https://BioRender.com/f3thf90, accessed on 10 September 2025.

Brain aging represents the convergence of multiple molecular and cellular pathways that progressively undermine neuronal integrity, synaptic plasticity, and network resilience (Figure 2). These changes are not isolated phenomena; rather, they interact in a cycle of inflammation, metabolic inefficiency, oxidative stress, and impaired repair mechanisms. Together, they lower the threshold for cognitive dysfunction when the aging brain is exposed to stressors such as anesthesia and surgery. By examining the molecular biology of brain aging, we can gain a deeper understanding of the origins of PNDs and target interventions to enhance resilience.

### 3.1. Aging and Neuroinflammation

Neuroinflammation is a central hallmark of the aging brain. Microglia and astrocytes shift over time toward a pro-inflammatory and less flexible phenotype. This chronic state, often referred to as inflammaging [29], creates a baseline vulnerability where even minor perturbations can trigger amplified neuroinflammatory cascades and maladaptive synaptic pruning.

Experimental studies demonstrate that aging microglia adopt a primed phenotype characterized by exaggerated cytokine release, increased oxidative stress, and reduced phagocytic capacity [30]. For example, microglia from middle-aged mice challenged with lipopolysaccharide show significantly greater induction of Il1b, Il6, and Tnf compared with those from young animals [31]. Aged hippocampal microglia also exhibit reduced phagocytosis of apoptotic newborn neurons, impairing neurogenesis [32]. Histological studies in aged rodent brains further corroborate these changes, showing increased Iba1+ microglial density and GFAP+ astrocytic hypertrophy in hippocampal and cortical regions [33,34]. The concept of microglial priming has been further supported by mechanistic studies demonstrating that aging sensitizes microglia to secondary stressors, leading to amplified pro-inflammatory responses [35]. Translational evidence from human postmortem studies shows that aged microglia accumulate lipofuscin, display increased oxidative stress markers, and exhibit impaired clearance functions [36]. Together, these changes contribute to inefficient clearance of amyloid-β and propagation of chronic neuroinflammation.

Systemic inflammatory markers in humans parallel these experimental findings. Older adults with higher perioperative plasma interleukin-6 (IL-6) and IL-8 are at greater risk of developing postoperative delirium [37]. In addition, the neutrophil-to-lymphocyte ratio has emerged as a practical biomarker of inflammaging, with elevated preoperative values independently associated with delirium after spine surgery in elderly patients [38]. These blood-based measures provide a peripheral window into neuroimmune vulnerability.

Central nervous system markers further strengthen the link between inflammation and aging. Cerebrospinal fluid (CSF) studies in delirious patients show elevated pro-inflammatory cytokines such as IL-1β, directly implicating central nervous system (CNS) inflammation in acute cognitive dysfunction [39]. In vivo imaging complements these findings: PET studies with [^11C]-PBR28 demonstrate that microglial activation correlates with reduced white-matter integrity and diminished network efficiency, independent of amyloid or atrophy [40]. Tau pathology, referring to the abnormal aggregation of microtubule-associated tau protein, adds an additional dimension, with microglial activation and tau independently predicting cognitive decline [41]. Functional MRI studies show that higher inflammatory burden disrupts resting-state connectivity within hippocampal and frontal networks essential for memory and executive function [42].

Together, evidence from animal histology, circulating blood markers, and central inflammatory indices—such as cerebrospinal fluid cytokine levels, PET imaging of microglial activation, and postmortem glial immunohistochemistry—converge to show that neuroinflammation is a defining feature of brain aging.

### 3.2. DNA Damage and Telomere Attrition

Alongside inflammation, genomic instability plays a major role in aging. Telomeres, protective DNA caps, progressively shorten with successive cell divisions. Oxidative stress has been consistently associated with accelerated telomere attrition, but causality remains uncertain. Collectively, evidence indicates that oxidative stress and telomere biology are interacting but distinct contributors to cellular senescence [43]. In the brain, shortened telomeres in hippocampal progenitors reduce proliferation and downregulate neurogenesis-associated genes, impairing the generation of new neurons [44]. Both animal and human studies support the link between telomere attrition and cognition: telomerase-deficient mice exhibit impaired hippocampal neurogenesis with associated memory deficits [45], while in older adults, shorter leukocyte telomere length has been associated with poorer cognitive performance [46]. Because hippocampal neurogenesis underlies learning and memory, telomere attrition may directly contribute to diminished cognitive reserve.

The aging human cortex also accumulates oxidative DNA damage at promoter regions of downregulated genes, particularly those involved in synaptic and mitochondrial function [47]. These include synaptic and calcium-signaling genes (e.g., CALM1, CAMK2A, PRKCG, VAMP1, SCN2B, SORT1), calcium homeostasis genes (ATP2B2, CALB1, CALB2), and mitochondrial oxidative phosphorylation components (ATP5A1) [47]. Critically, base excision repair (BER) capacity declines with age, and additional pathways are also impaired. Nucleotide excision repair (NER) shows reduced activity in aged fibroblasts, mismatch repair efficiency decreases in aged stem cells, and double-strand break repair (both non-homologous end joining and homologous recombination) exhibits diminished fidelity with age [48,49]. BER gene expression remains positively correlated with brain-derived neurotrophic factor (BDNF) signaling, which itself declines with aging [50].

Together, these findings highlight a brain with shrinking genomic defenses. This creates lasting transcriptional deficits that may manifest as postoperative cognitive decline. Importantly, these data also suggest that interventions boosting BDNF or repair capacity—such as exercise or pharmacological agents—could reduce perioperative vulnerability.

### 3.3. Oxidative Stress and Mitochondrial Dysfunction

Oxidative stress is a unifying feature of aging and neurodegeneration. ROS are byproducts of cellular respiration, but in aging brains, mitochondrial inefficiency leads to overproduction of ROS while simultaneously reducing antioxidant defenses. This imbalance promotes DNA, lipid, and protein damage, and reinforces amyloid and tau accumulation [51]. Oxidative stress also directly impairs synaptic proteins, vesicle trafficking, and calcium regulation, leading to synaptic dysfunction even before neuron loss occurs [52].

Mitochondria are both the source and victim of this process. With age, they produce less ATP, handle calcium less effectively, and release more ROS, creating a vicious cycle of metabolic and structural decline. Experimental studies show that mitochondria isolated from aged rodent brains generate more ROS at respiratory complex I compared with young animals [53], while analyses of human brain tissue demonstrate increased protein oxidation and lipid peroxidation, consistent with insufficient clearance of ROS and progressive redox imbalance [54]. Beyond direct oxidative injury, ROS also induces maladaptive epigenetic modifications, shifting gene expression toward pro-apoptotic and inflammatory programs.

### 3.4. Metabolic Change

Metabolic decline is increasingly recognized as an upstream driver of brain aging. Energy surplus—chronic caloric intake, insulin resistance, and obesity—accelerates brain aging by amplifying oxidative stress, inflammation, and protein aggregation, while intermittent metabolic challenges such as caloric restriction and exercise enhance resilience [55].

Normal aging induces metabolic inefficiency across multiple domains, including mitochondrial energy metabolism, neurotransmitter balance, and calcium regulation [56]. A metabolomic atlas of the aging mouse brain has revealed region-specific alterations in lipid and energy pathways and reduced interregional metabolic coordination [57]. This suggests that some brain regions (e.g., hippocampus, frontal cortex) may be metabolically more fragile than others. In humans, reduced glucose metabolism on PET precedes structural atrophy and predicts cognitive decline [58].

### 3.5. Synaptic Architecture and Neurotransmission

Synaptic changes are among the strongest correlates of cognitive decline in aging. Aging selectively reduces thin, plastic dendritic spines in prefrontal and hippocampal neurons—precisely the synapses most critical for learning and memory [59]. Loss of these spines correlates more closely with cognitive decline than does overall neuronal loss, underscoring the primacy of synaptic integrity over sheer neuron number. Nevertheless, neuron density itself also declines in vulnerable regions such as the hippocampus and prefrontal cortex, reducing overall connectivity and network reserve [60].

Age-related impairments in neuroplasticity further compound these structural changes. Long-term potentiation (LTP), a cellular correlate of learning and memory, is diminished in the aged hippocampus, reflecting deficits in calcium signaling, actin cytoskeletal remodeling, and trophic factor support. Parallel declines in neurogenesis limit the brain’s capacity to replace or remodel circuits in response to injury or environmental demands.

In addition, myelination is increasingly recognized as a critical factor in cognitive aging. Human neuroimaging studies show that reduced myelin content in frontal and temporal regions correlates with poorer memory performance in healthy older adults, supporting the structural disconnection hypothesis [61]. Such myelin vulnerability likely interacts with synaptic changes to amplify deficits in connectivity and information processing.

Oxidative stress accelerates synaptic instability, disrupting protein turnover and spine morphology [62]. These data suggest that vulnerability in the aging brain is multifactorial. Neuroinflammation, oxidative stress, and metabolic dysfunction converge not only on synapses but also impair myelin and axonal function. Together, these disruptions destabilize neural networks and heighten susceptibility to anesthetic exposure, helping explain the strong link between structural decline and perioperative cognitive dysfunction.

## 4. Effect of Anesthesia on the Aging Brain

Due to the significant burden of PNDs, especially in aging patients with less cognitive reserve, current studies aim to further understand the mechanisms behind surgery/anesthesia exposure and the development of PNDs. It is believed that neuroinflammation, mitochondrial dysfunction, alterations in synaptic function/plasticity, and disruption of the glymphatic system are significant contributors to the development of PNDs. Peripheral inflammation during anesthesia/surgery can induce neuroinflammation through multiple mechanisms such as: entering the CNS through the periventricular zone or active transport, the vagus nerve pathway, stimulating brain vascular endothelial cells to secrete pro-inflammatory cytokines into the CNS, disruption of the blood–brain barrier (BBB), and the activation of glial cells [63]. Importantly, surgical stress amplifies the vulnerability of aging patients primed to pro-inflammatory states: in murine models, peripheral surgery induces hippocampal microglial activation, elevated Interleukin (IL)-1β and Tumor necrosis factor-alpha (TNF-α), synaptic loss, and subsequent memory impairment [64]. Such histological evidence underscores that age-related neuroinflammatory priming translates into measurable structural and cognitive consequences.

### 4.1. Anesthesia and Neuroinflammation

Even with the BBB remaining intact, neuroinflammation can result by homologous receptors on BBB endothelial cells being stimulated by IL-1 resulting in the release of pro-inflammatory mediators such as TNF-ɑ, IL-6, IL-1 and Prostaglandin E2 (PGE2) into the brain parenchyma following surgery [65]. In regard to the vagus nerve pathway, in a study by Yang et al. in which mice underwent laparotomy, excessive glutamate release by vagal nerve terminals activated brain mast cells by binding to the NR2B subunit of NMDA receptors. The brain mast cells activated glial cells resulting in the release of pro-inflammatory mediators and promoted cell apoptosis [66]. The release of TNF-ɑ by brain mast cells also promotes recruitment of neutrophils and BBB disruption [67].

Hu et al. further demonstrated that in aged mice underwent laparotomy under 3% sevoflurane, peripheral inflammation activated Matrix Metalloproteinase-9 (MMP9) which resulted in pericyte injury, tight junction breakdown and ultimately BBB disruption. The impaired BBB resulted in the propagation of CNS inflammation, synaptic dysfunction and behaviors of post-op delirium. MMP9 inhibitor (SB-3CT) diminished pericyte and tight junction injury, preserved BBB function, and minimized neuroinflammation. This ultimately reducing synaptic dysfunction and post-op delirium behavior. This effect was also seen by the administration of melatonin due to its ability to act as an endogenous inhibitor of MMP9 [68].

Moreover, in an animal study involving rats by Xu et al., compared to controls after prolonged sevoflurane exposure, pro-inflammatory cytokines including TNF-ɑ, IL-1B, and IL-6 were increased in the hippocampus via the NF-kb pathway. Additionally, anti-inflammatory markers including IL-4, IL-10, CD206, and Arginase-1 were found to be downregulated. It was also found that the number of microglia increased in the hippocampus after sevoflurane exposure compared to the control group. The activated microglia induce neuroinflammation and complement activation, engulf synapses and cause synaptic loss contributing to cognitive deficits [69]. To further investigate the role of inflammation in PNDs, Xu et al. used Meloxicam to inhibit hippocampal neuroinflammation and found complement cascades, microglial activation and ultimately synaptic loss were interrupted. They also found that the use of PLX3397 (a selective colony-stimulating factor 1 receptor inhibitor that has been demonstrated to readily cross the BBB and deplete microglia [70], reversed synaptic elimination, cognitive impairment and anxiety behaviors caused by prolonged anesthesia.

Xu et al. also found that sevoflurane administration reduced neuronal excitability in the hippocampal CA1 region (a region important for learning and memory) and resulted in burst suppression in the hippocampus on EEG. Similarly, in regard to burst suppression, a cohort study by Fritz et al. involving adult patients receiving general anesthesia showed that patients under anesthesia with more burst suppression on EEG were more likely to experience delirium compared to patients with no burst suppression [71]. Patients with higher end-tidal volatile anesthetic concentration were more likely to experience EEG suppression [71].

### 4.2. Mitochondrial Impact

Studies have explored how surgery/anesthesia disrupts mitochondrial function. Previous studies have found that mitochondrial fission/fusion dynamics play a significant role in synaptic plasticity, neurotransmitter synthesis and degradation, ROS production and sequestration, and apoptosis [72]. With the high aerobic metabolism of the brain, the brain is also more vulnerable to oxidative injury caused by ROS. Due to the important role mitochondria play in cognitive function, studies have explored how surgery/anesthesia affect mitochondrial function. Perioperative stressors such as anesthesia and surgery can exacerbate oxidative imbalance, contributing to systemic and neurological complications [73]. It has been found that volatile anesthetics as well as phenobarbital and propofol can dose-dependently inhibit mitochondrial respiration [74]. In a study in which aged mice underwent laparotomy under 1.4% isoflurane anesthesia for 2 h, it was found that surgery/anesthesia disrupted mitochondrial function in the brain of the aged mice resulting in excessive accumulation of ROS, impaired superoxide dismutase activity, defects in energy metabolism with reduced mitochondrial membrane potential and ATP production with consequent cognitive dysfunction [75].

### 4.3. Synaptic Function and Neuroplasticity

Prolonged anesthesia itself has been shown to alter synaptic architecture and impair object-recognition memory in mice [76]. For instance, with excessive oxidative stress and mitochondrial dysfunction Lu et al. also found that there was decreased expression of neuronal/synaptic plasticity related proteins such as Postsynaptic Density Protein-95 (PSD-95) and BDNF after exposure to surgery/anesthesia [75]. This is important because it was found that neuronal/plasticity related proteins such as PSD-95 and BDNF were involved in synaptic transmission and play an important role in learning and memory [77]. BDNF is also crucial for the development and plasticity of glutamatergic and GABAergic synapses [78]. Thus, surgery/anesthesia can lead to mitochondrial dysfunction, resulting in excessive oxidative stress and energy deficits and the downregulation of important neuronal/synaptic plasticity related proteins ultimately playing a role in the development of PNDs. 

Additionally, another study by Wu et al. found that older mice had reduced Sirtuin 1(SIRT1)/BDNF expression, impaired synaptic plasticity and decreased neuronal excitability in glutamatergic neurons after anesthesia/surgery which was accompanied by significant cognitive impairment postoperatively [79]. SIRT has been shown to have neuroprotective effects in central neurodegenerative diseases and a role in memory formation by regulating synaptic plasticity [80,81], thus highlighting how SIRT’s downregulation with surgery/anesthesia exposure can contribute to PNDs. Additionally, surgery/anesthesia was found to reduce synaptic plasticity through inhibiting autophagy. Autophagy and its regulation by mTOR are critical for maintaining functions such as synaptic remodeling and plasticity [82]. Gao et al., 2021 found that surgery/anesthesia induced hyperactivation of mTOR and decreased autophagy reducing neuronal/synaptic plasticity related proteins such as synaptophysin and PSD-95 which resulted in memory and cognitive impairment [83].

### 4.4. Anesthetic Effects on RNA

The effect of anesthesia exposure on long noncoding RNAs (lncRNAs) is an additional mechanism explored in its involvement in the development of PNDs. LncRNAs are noncoding RNAs which play significant roles in the regulation of tissue homeostasis, oxidative stress, and metabolism [84,85]. Differential expression and dysregulation of lncRNAs have been linked to neurologic disorders such as schizophrenia, Alzheimer’s disease, and autism spectrum disorder [86,87]. In an animal study involving aged mice, sevoflurane exposure was found to be associated with differentially expressed lncRNAs involved in processes such as neurologic system alteration, neuronal development, metabolism alteration, immunity and neuroinflammation, apoptosis and autophagy, cellular communication, molecular modification and behavioral changes [88]. For instance, sevoflurane exposure was found to be associated with a decreased expression of MALAT1 in the hippocampus of aged mice [88]. MALAT1 is one of the most highly expressed lncRNAs in the CNS and has a crucial role in neuronal development [89]. Additionally MALAT1 suppresses neuronal apoptosis and neuroinflammation [90]. Via lncRNAs, sevoflurane exposure also altered the expression of mRNAs such as: Dlg4 (important for synaptic function), Avp (important for lipophagy), Islr2 (important for synaptic function), Hcrt (regulates sleep–wake behavior), and Tnc (involved in neurotransmitter uptake) [88]. Moreover, the study by Chen et al. found that a large number of lncRNAs related to the apoptosis pathway were differentially expressed in the hippocampus after sevoflurane exposure [91]. They found that sevoflurane exposure up-regulated lncRNA, inducing overexpression of Bcl2l11(BIM) and ultimately promoting mitochondria-mediated apoptosis. They also found a reduction in anti-apoptotic proteins Bcl-2 in the hippocampus [91]. Previous studies have found that over-expression of BIM can induce apoptosis by activation of the pro-apoptotic protein Bax or neutralization of Bcl-2 [92]. In another study, Qu et al. found differential expression of 3 main lncRNAs which they termed sevoflurane associated noncoding RNA (Sancr) in the hippocampus of aged rats after sevoflurane exposure. They found that the Sancrs are associated with mitochondrial dysfunction, oxidative stress, DNA damage, apoptosis, and neurodegenerative features in the hippocampus [93].

### 4.5. Anesthetic Choice and PNDs

When evaluating the risk of anesthesia and PNDs, it is also important to consider which medications are utilized during anesthetic management. Volatiles are thought to induce pro-inflammatory states, downregulate anti-inflammatory markers, disrupt mitochondrial function inducing oxidative stress, and cause synaptic dysfunction [59,65,69].

Propofol was found to disrupt the integrity of synapses and increase apoptosis in developing brain neurons and immature astrocytes [94]. Propofol was also found to disrupt mitochondrial function and increase ROS in neurons [95].

A study exploring the incidence of post-op delirium in fentanyl vs. remifentanil use in general anesthesia found the incidence of delirium in the recovery room to be 12.2% for fentanyl vs. 7.7% for remifentanil and 5.8% vs. 1.9% on the first postoperative day [96]. It is theorized that dexmedetomidine and sufentanil may lower incidence of PNDs through dampening surgical stress response. For example, dexmedetomidine promotes the secretion of anti-inflammatory factor IL-10 and inhibits the production of TNF-α and IL-1β, in this way it adjusts the balance of systemic proinflammation and anti-inflammation. Dexmedetomidine acts on α2 adrenoceptors in the central and peripheral nervous system, inhibiting sympathetic activity, reducing stress response, and activating cholinergic anti-inflammatory pathways, thus attenuating the intensity of systemic inflammatory response [97]. Similarly, it has been shown that sufentanil can inhibit the release of inflammatory factors and reduce the stress response. In a rat study involving pretreatment with sufentanil in cerebral ischemia–reperfusion injury, sufentanil reduced the expression of inflammatory cytokines including interleukin IL-1B, IL-4, IL-6, IL-8, IL-10, and TNF-ɑ. Sufentanil also inhibited MMP2, MMP9, and promoted the expression of collagen IV which indicated that sufentanil attenuated the destruction of the BBB [98]. Additionally, a study evaluating the incidence of POCD in older adults undergoing open surgery with general anesthesia and either sufentanil or fentanyl use found the incidence of POCD to be 6.2% in the sufentanil group vs. 11.9% in the fentanyl group [99].

Additionally, studies investigated the risk of POD when choosing between regional or neuraxial anesthesia vs. general anesthesia. For example, a randomized control trial looking at the effect of regional anesthesia without sedation vs. general anesthesia on the incidence of POD in older patients undergoing hip fracture surgery found no significant difference between the groups [100]. Similarly, Cheung et al. performed a meta-analysis looking at the risk of POD in elderly patients undergoing hip fracture surgery under either neuraxial anesthesia or general anesthesia and found there was no significant difference in the incidence of POD between the two techniques [101].

An understanding of the pathophysiology behind PNDs and its risk factors are crucial for identifying patients at higher risk for development of PNDs. As clinical context allows, choice of anesthetic agent may have a role in managing patients at significant risk of PNDs. Postoperatively, additional care should be taken in identifying the development of PNDs and promptly treating it in order to reduce the duration and severity of cognitive dysfunction.

### 4.6. Anesthesia and the Glymphatic System

The disruption of the glymphatic system is also being evaluated in its role in aging, neurodegenerative diseases, and alterations during anesthesia exposure. With the high metabolic activity of the brain, neurons in the brain require rapid elimination of metabolic byproducts to maintain homeostasis and a physiologically normal environment. The glymphatic system plays a critical role in the elimination of cerebral metabolites and toxins using CSF [102,103]. The dysfunction of this system has been implicated in age-associated cognitive decline and neurologic diseases including Alzheimer’s disease and multiple sclerosis [104]. In animal studies, sevoflurane and isoflurane were found to decrease the circulatory function of the glymphatic system via aquaporin-4 (AQP4) depolarization and result in the accumulation of metabolic waste products and an increase in the levels of inflammatory proteins [105,106,107]. This accumulation of metabolic waste products was found to damage neurons and synapses and lead to a decline in learning and memory in animal studies. One mechanism through which this occurred was by the accumulation of Aβ and Tau which downregulated the expression of PSD-95 in the hippocampus [94,106]. A more recent human study also found that glymphatic function was not only reduced while patients were under anesthesia with sevoflurane but also 45 min after emergence [96,108]. Enhanced AQP4 polarization could attenuate cognitive dysfunction in mice by suppressing neuroinflammation and enhancing glymphatic function thus acting as a potential treatment target for PNDs [105]. Additionally, dexmedetomidine could improve glymphatic function and have protective effects against sevoflurane-induced damage to the circulatory function of the glymphatic system [106]. Ketamine/Xylazine was also found to have potential increases in the glymphatic system activity [107]. However, these findings are conflicting as a newer study suggests that although glymphatic influx of CSF and metabolic waste may increase, brain clearance of metabolites and toxins from the brain may actually be reduced even by dexmedetomidine and ketamine/xylazine [109].

## 5. Molecular and Clinical Targets for PND Management

PNDs represent a spectrum of cognitive impairments that frequently complicate the postoperative course of older adults. This continuum, encompassing POD and long-term cognitive decline, is associated with increased morbidity, mortality, and healthcare costs. The pathophysiology is understood to be multifactorial, with neuroinflammation emerging as a central theme. However, the precise mechanisms linking surgery and anesthesia to cognitive decline remain an area of intense investigation, with a landscape of often conflicting clinical evidence. Identifying treatment targets for PND management is crucial in order to reduce its incidence and severity and mitigate the burden of morbidity, mortality, and healthcare cost.

### 5.1. The Molecular Cascade of Neuroinflammation

Given the central role of neuroinflammation in both brain aging and the acute stress of surgery, this pathway presents a primary clinical target. The surgical trauma itself triggers a systemic inflammatory response that can overwhelm the defenses of an aging brain, which is often already in a primed, pro-inflammatory state [110]. This neuroinflammatory cascade is compounded by a second, related vulnerability: the impairment of the brain’s waste clearance. The glymphatic system, responsible for clearing metabolic byproducts, is highly dependent on sleep and can be suppressed by certain anesthetic agents such as volatile anesthetics and improved with dexmedetomidine [111]. The subsequent accumulation of neurotoxic waste, exacerbated by postoperative sleep disruption, offers another clear mechanism for intervention [112]. Therefore, strategies aimed at either directly mitigating the inflammatory response or preserving the brain’s natural restorative and clearance processes form the basis of modern PND management.

### 5.2. Dexmedetomidine

**Figure 3:**
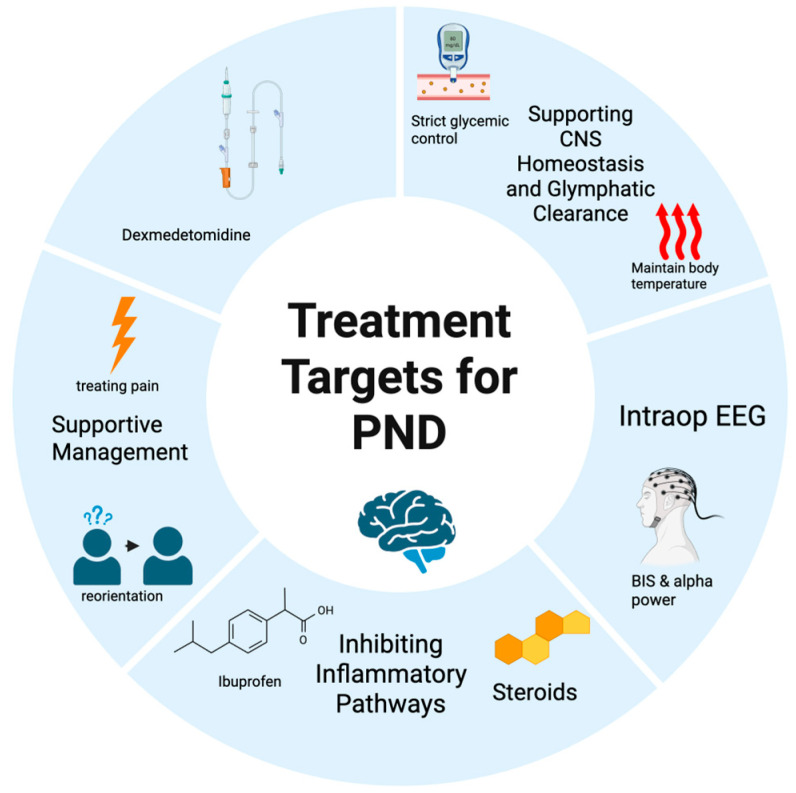
Treatment Targets for PND. Created in BioRender. Tawfik, G. (2025) https://BioRender.com/4fopoik, accessed on 10 September 2025.

This highly selective alpha-2 adrenergic receptor agonist has garnered significant attention for its potential neuroprotective qualities (Figure 3). Its mechanisms of action are thought to include the attenuation of the surgical stress response and a reduction in neuroinflammation. A substantial body of evidence from multiple meta-analyses supports its use. One systematic review from 2019 concluded that dexmedetomidine is a safe and effective agent for preventing POD in elderly patients after non-cardiac surgery [113]. More recent data reinforce this, with a 2025 meta-analysis of 10 trials (2286 patients) finding that perioperative dexmedetomidine sedation was associated with a 47% relative risk reduction for POD in adults undergoing non-cardiac surgery. Similar protective effects have also been observed in patients undergoing cardiac surgery [114].

The effectiveness of dexmedetomidine appears to extend across the different motor subtypes of delirium. Delirium can manifest in various psychomotor subtypes, including hyperactive, hypoactive, and mixed forms [115]. The hypoactive subtype, characterized by lethargy and confusion, often goes unrecognized and is associated with a poorer prognosis [116]. A 2024 randomized trial found that intraoperative dexmedetomidine significantly reduced the incidence of hypoactive delirium from 28.5% in the control group to 5.7% in the treatment group [117]. Furthermore, other evidence suggests that low-dose dexmedetomidine can reduce the incidence of all three motoric subtypes of delirium, making it a broad-spectrum prophylactic agent [118].

Crucially, the dosing strategy appears to be a key determinant of efficacy. The non-cardiac surgery meta-analysis found that low-dose infusions (less than 0.2 µg/kg/h) were more effective, reducing the relative risk by 62%, compared to a 34% reduction with higher doses. This suggests the benefit may stem from the drug’s anti-inflammatory and stress-reducing properties rather than its sedative effects, which are more prominent at higher doses. However, the clinical application of dexmedetomidine is not without nuance. The large SPICE III randomized clinical trial, which investigated its use for sedation in critically ill patients, found that while it had a similar mortality rate to usual care, it was associated with a higher rate of bradycardia and hypotension [119]. This highlights that while low-dose dexmedetomidine may be beneficial for delirium prevention in a stable perioperative population, its hemodynamic profile requires careful consideration.

### 5.3. Non-Steroidal Anti-Inflammatory Drugs (NSAIDs)

Given the inflammatory basis of PNDs, targeting inflammation with cyclooxygenase inhibitors seems a logical therapeutic strategy. A 2024 meta-analysis of eight randomized controlled trials involving 1238 participants associated the perioperative administration of NSAIDs with a reduced risk of POD, lowering the incidence from 19% in control groups to 11% in the NSAID groups [120]. A separate large-scale database analysis further specified that non-salicylate NSAIDs (e.g., ibuprofen, ketorolac) were more effective than aspirin or acetaminophen. While these findings are promising, they must be interpreted with considerable caution. The substantial and well-documented risks of NSAIDs in older adults, including renal injury and gastrointestinal bleeding, cannot be overlooked. Therefore, the decision to use NSAIDs must be highly individualized, carefully balancing the potential for delirium prevention against the clear risk of significant harm.

### 5.4. The Role of EEG Guidance

The hypothesis that excessively deep anesthesia contributes to PNDs has driven the adoption of EEG-based depth of anesthesia monitors. The goal is to avoid periods of excessive cortical suppression, which are thought to be neurotoxic. An EEG pattern known as burst suppression—periods of electrical silence—is a marker of this profound inactivation and has been associated with an increased risk of POD. Similarly, lower power in the alpha frequency band (8–12 Hz) on the intraoperative EEG has also been identified as a potential marker for a vulnerable brain and is associated with a higher risk of delirium [121,122].

A 2018 systematic review and meta-analysis identified 5 studies which reported postoperative delirium in association with processed EEG-guided anesthesia. The authors reported that processed EEG use was associated with a statistically significant reduction in POD [123]. On the other hand, the ENGAGES randomized clinical trial, a landmark study involving over 1200 patients undergoing major surgery under general anesthesia, did not reduce the incidence of POD compared to routine practice [124]. Possible explanations for this inconsistency include publication bias which limits the reliability of meta-analyses, or unidentified confounders in prior studies.

Another consideration is that not all anesthetic-induced states of unconsciousness are neurophysiologically equivalent. While the neuroprotective effects of an agent like dexmedetomidine are multifactorial, its unique EEG signature is a key area of investigation. Unlike GABAergic agents such as propofol, dexmedetomidine induces an EEG pattern that has been quantitatively validated by machine learning to be homologous to natural NREM Stage 3 sleep, characterized by sleep spindles and slow-wave activity [125]. This distinction is clinically relevant, as specific sleep-like EEG features are directly correlated with PND outcomes. For instance, an EEG trajectory during emergence from anesthesia that lacks significant spindle activity is associated with a higher incidence of PNDs, and abrupt transitions from slow-wave states to wakefulness are linked to emergence delirium [126]. Dexmedetomidine appears to promote a more stable and organized emergence, potentially explaining some of its neuroprotective effects [127].

### 5.5. Future Directions

The management of PNDs remains a significant clinical challenge characterized by conflicting evidence. Pharmacological interventions like low-dose dexmedetomidine show consistent promise for delirium prevention but require careful patient selection. Technological interventions like EEG guidance have not proven to be a definitive solution, suggesting a more sophisticated understanding of intraoperative brain dynamics is needed. Future research should focus on prospectively testing whether anesthetic techniques that preserve or mimic the restorative neurophysiology of natural sleep can directly improve cognitive outcomes. Elucidating the role of the glymphatic system in clearing neurotoxic waste during these sleep-like states is another critical area of inquiry that could further explain the link between specific anesthetic choices and brain health [128].

## 6. Conclusions

When caring for patients perioperatively, providers must be mindful of the risk factors for and significant impact of PNDs. Although most often seen in elderly patients, PNDs can affect patients across all ages. There is often a lack of systemic evaluation of preoperative and postoperative neurocognitive disorders which may prevent or delay care; however, PNDs can undermine patient autonomy, have deleterious effects on quality of life, increase morbidity and mortality and add additional burden to hospital resources and cost of care. Understanding the risk factors and mechanisms of a PND is crucial in order for providers to take necessary steps to diminish the risk for and severity of it. Furthermore, understanding the mechanisms behind brain aging and the parallels to the pathophysiology of PNDs such as neuroinflammation involving oxidative stress, mitochondrial dysfunction and synaptic disruption are also integral to identify key targets in mitigating the incidence and severity of PNDs. Current research suggests possible clinical targets for management such as dexmedetomidine and NSAIDs due to their abilities to combat the harmful neuroinflammatory effects which is a key driver in the development of PNDs. Moreover, when providing care, EEG-guided anesthesia, careful choice of anesthetics, and supportive measures also can be utilized to aid in mitigating PNDs. It is important to note that much of our understanding of the pathophysiology behind PNDs is derived from animal studies; therefore, these findings may not fully represent the effects observed in patients in real-world clinical situations. Further research is also needed in order to identify and evaluate additional molecular and clinical targets for PND management. In this way, we can further our understanding of PNDs and better tailor our management in order to attenuate its incidence and severity.

## Data Availability

No new data were created or analyzed in this study. Data sharing is not applicable to this article. Studies cited in this review may be found in the References section.

## Abbreviations

The following abbreviations are used in this manuscript:

AQP4	Aquaporin-4
BBB	Blood–Brain Barrier
BDNF	Brain-derived Neurotrophic Factor
BER	Base Excision Repair
BIM	Bcl2l11
CNS	Central Nervous System
CSF	Cerebrospinal Fluid
dNCR	Delayed Neurocognitive Recovery
IL	Interleukin
lncRNAs	Long Noncoding RNAs
LTP	Long-term Potentiation
MMP9	Matrix Metalloproteinase-9
NCD	Neurocognitive Dysfunction
NSAIDs	Non-Steroidal Anti-Inflammatory Drugs
PGE2	Prostaglandin E2
PND	Postoperative Neurocognitive Disorders
POCD	Postoperative Cognitive Dysfunction
POD	Postoperative Delirium
PSD-95	Postsynaptic Density Protein-95
ROS	Reactive Oxygen Species
Sancr	Sevoflurane Associated Noncoding RNA
SIRT1	Sirtuin 1
TNF-α	Tumor Necrosis Factor-Alpha
